# Caregivers’ experience of having a child with Down syndrome: a meta-synthesis

**DOI:** 10.1186/s12912-024-02652-y

**Published:** 2025-01-20

**Authors:** Xiao Nan Zhang, Shuo Zhang, Chun Yan Liu, Zhi Hong Ni, Hai Tao Lv

**Affiliations:** 1 Department of Nursing, Children’s Hospital of Soochow University, No. 92 Zhong Nan Street, Soochow, Jiangsu Province China; 2 School of Nursing, Medical College of Soochow University, No. 1 Shi Zhi Road, Soochow, Jiangsu Province China; 3 Children’s Hospital of Soochow University, No. 92 Zhong Nan St, Suzhou, 215025 China

**Keywords:** Down syndrome, Trisomy 21 syndrome, Chromosomal disease, Caregiver stress, Meta-synthesis

## Abstract

**Background:**

This study aimed to integrate the experiences of caregivers of children with Down syndrome during the care process and understand their feelings and needs.

**Methods:**

We used Page et al.’s (2021) Preferred Reporting Items for Systematic Reviews and Meta-synthesis Statement. Ten databases (Web of Science, PubMed, EMBASE, Cochrane Library, CINAHL, PsycInfo, China Biology Medicine, China National Knowledge Infrastructure, Wanfang Data, and China Science and Technology Journal Database) were searched for relevant studies published from the inception of the database to October 2023. Eight qualitative studies were analysed. The following seven themes were included: ‘feeding pressure’, ‘hope for education’, ‘societal rejection and stigma’, ‘psychological pressure’, ‘caring burden’, ‘family burden’, and ‘family adaptation and self-growth’.

**Results:**

We found that feeding pressures, educational concerns, language difficulties, and discrimination and stigmatisation led to psychological, economic, and family stress in caregivers of children with Down syndrome. We document the need for strong coping mechanisms and support systems for these families from medical and psychological institutions and a need for public education and awareness.

**Conclusions:**

We summarised the daily care experiences of caregivers of children with Down syndrome. Our findings provide a scientific basis for further research focused on reducing physical and mental pressure on caregivers and improving the quality of family life.

**Supplementary Information:**

The online version contains supplementary material available at 10.1186/s12912-024-02652-y.

## Background

Down syndrome (DS), also known as trisomy 21 syndrome, is caused by a third copy of all or part of the chromosome HSA21 [1]. It is the earliest confirmed chromosomal disease in humans and the most common cause of developmental and intellectual disabilities in children. The older the mother, the higher is the incidence rate [2]. Children with DS have unique physical characteristics and face physical and mental health problems such as developmental delays, intellectual disabilities, and delayed development of motor and language skills [3].

The live birth rate of DS varies by country and region; the current estimated live birth rate of DS ranges from 1/546 to 1/1350 [4–7], making it the most common genetic cause of intellectual disabilities [5]. In 2018, the birth rate of children in China was 1.37 per 10,000 people [8]. Approximately 1 in every 700 live births in the United States is born with DS, and approximately 5,100 babies with DS are born each year [9]. In the United Kingdom, approximately one in every 444 live births is diagnosed with DS [10].

DS cannot be cured but can be improved through drug therapy [11], language intervention [12], exercise intervention [13], and other methods to enhance the quality of life of children with DS and their families. Drug therapy is commonly used to control various comorbid health conditions in patients with DS, e.g., methylphenidate is commonly used in DS with attention deficit hyperactivity disorder (ADHD), Cerebrolysin^®^ is used to improve cognitive development, and thyroid hormone and folic acid are used to improve psychomotor development [11], which leads to a high drug use rate in these patients. Caregivers of patients with DS may need to monitor adverse reactions [14], compliance, and treatment outcomes strictly when the patient is receiving medication. A more complete description of the factors affecting DS medication will help improve the clinical care of patients with DS [15]. Language interventions for children with DS strive to improve their communication as well as their academic, social, and professional functions [16]. Speech therapy is effective throughout childhood [17], and complications caused by DS, such as type I diabetes, obesity, hypotonia, or osteoarthritis, can be improved through specific sports programmes. Regular daily relaxation or stretching exercises can help patients control diabetes [18], resistance training can effectively improve muscle strength, and cardiovascular and strength training have a positive impact on BMI [19].

Owing to the unique nature of DS, caregivers of children with DS must take care of children who are cognitively, physically, and behaviourally different from their peers. Historical research has found that families of children with DS face multiple challenges related to intellectual disability, health, and sociopsychological development [20, 21]. Caregivers not only need to face the health problems caused by the disease itself but also must address a series of potential complications. For example, children with DS combined with swallowing difficulties are highly prone to aspiration [22]; children with DS combined with autism spectrum disorder (ASD) encounter many stereotypes and may experience social withdrawal and self-harm behaviours [23]; and sexual dysfunction in male patients with DS increases with age, leading to a loss of fertility [24]. Existing health issues and concerns about the future cause parents of children with DS to bear greater parenting pressure than parents of normal children [25, 26]. Stress can adversely affect the mental health and happiness of parents [27], placing heavy physical and mental burdens on caregivers and increasing the risk of mental health problems. Additionally, psychological pressure on parents can affect children’s health as well as their psychological and developmental outcomes [28]. Therefore, it is necessary to focus on the experiences of caregivers of children with DS, understand their stress and needs, and provide targeted assistance.

Dunn et al. [29] integrated the psychological health and happiness experiences of caregivers of children with developmental disorders. Masefield et al. [30] found that mothers of children with developmental disorders may have worse health than those of normally developing children. However, these meta-analysis neither distinguished between specific diseases nor included only English literature. There are few reports on the integration of qualitative research into the experiences of caregivers of children with DS. Our meta-synthesis focuses on children with DS (from birth to the age of 18) and delves into the experiences of caregivers for this disease. The research results are targeted and representative of caregiving for children with DS. In addition, we compared the experiences of caregivers of children with DS with those of children with other developmental disorders and found that caregivers of children with DS had higher feeding pressure than caregivers of children with other developmental disorders, providing a target for beneficial guidance from medical staff in the later stage. Caregivers included the child’s parents, grandparents, maternal grandparents, and brothers. When collecting literature, we carefully chose studies to represent as diverse a sample of caregivers as possible.

### Aim

This study aimed to examine the experiences and feelings of caregivers of children with DS in their daily care process. We also aimed to understand the difficulties faced by caregivers of children with DS, how they responded, and their needs. Our findings will help clinical doctors and nurses understand the experiences and challenges of these caregivers, provide emotional support and psychological counselling, and provide accurate DS-related information to families of children with DS. These results can help meet caregivers’ needs and reduce their care burden, and serve as a basis for policymaking and guidance in clinical practice.

## Methods

### Design

Meta-synthesis is a research method that reviews and analyzes qualitative research results related to a phenomenon to generate a new comprehensive understanding of the topic [31]. This study was conducted under the guidance of Purssell et al. [32], following the preferred reporting items of the system evaluation and meta-synthesis (PRISMA) checklist guidelines [33]. A Critical Assessment Skills Program (CASP) for Qualitative Research was used to evaluate the methodological quality of research [34]. A content topic analysis method [35] was used to encode and group the qualitative research results.

### Search methods

With the assistance of professional librarians, the databases Web of Science, PubMed, EMBASE, Cochrane Library, CINAHL, PsycInfo, China Biology Medicine, China National Knowledge Infrastructure, Wanfang Data, and the China Science and Technology Journal Database were used. The search period was from inception of the database to October 2023. We selected search terms from the list of MESH search terms and conducted a comprehensive literature search using a combination of topics and free words. The representative search terms used in the search were ‘Down syndrome’, ‘Caregivers’, ‘Experience’, and ‘Qualitative Research’ from the list of Medical Subject Headings (Mesh terms). The search terms were used with the Boolean operators ‘AND’ and ‘OR’ for multiple combination searches and to trace the references included in the studies. The strategies used in this study are presented in Table S1 in the Supplementary Material.

The inclusion criteria were as follows: (a) the research participants were documented as caregivers of children with DS, with normal cognitive and communication abilities, including parents or relatives, regardless of age; (b) children aged 0–18 years and diagnosed with DS; (c) research on the feelings, experiences, parenting adaptation, and coping strategies of caregivers of children with DS; and (d) the primary research methods were qualitative research, including phenomenological research, grounded theory, and exploratory research. The exclusion criteria were as follows: (1) the research object was unclear; (2) the research participants included unrelated populations, such as medical staff; (3) inability to extract effective information from the literature; (4) inability to obtain the full text; and (5) non-qualitative research or unclear research methods.

After removing duplicate articles, the first author screened titles and abstracts based on the inclusion and exclusion criteria. Articles that did not meet the inclusion criteria were excluded. The remaining articles were screened, selected, and rigorously evaluated by two reviewers (ZXN and ZS) for the selected research. When a reviewer raised objections, disagreements were resolved by a third expert with evidence-based experience.

### Quality appraisal

The JBI Key Assessment checklist [36] was used to evaluate the methodological quality of the study. There are a total of 10 evaluation indicators, with the results being ‘yes’, ‘no’, ‘unclear’, and ‘not applicable’. If a study reports ‘yes’ in eight or more questions, it is classified as Class A, with a low likelihood of bias; if six or seven questions are reported as ‘yes’, it is classified as Class B and there is a possibility of moderate bias; and if a study reports five questions or less as ‘yes’, it is classified as Class C, with a high likelihood of bias. Only studies classified as A or B were considered for this meta-synthesis. The results of the quality evaluation are listed in Table 1. In case of disagreement in the evaluation opinions of the two researchers, it was decided upon by members of the research group through discussion.

**Table 1:** Methodological quality appraisal of included studies (*n* = 8)

Article codes	Author (year)	Q1	Q2	Q3	Q4	Q5	Q6	Q7	Q8	Q9	Q10	Grade
(A1)	Zhang (2017) [37]	Y	Y	Y	Y	Y	Y	N	Y	U	Y	A
(A2)	Çelik and Uzun (2023) [38]	Y	Y	Y	Y	Y	N	Y	Y	Y	Y	A
(A3)	Ridding and Williams (2019) [39]	Y	Y	Y	Y	Y	N	Y	Y	Y	Y	A
(A4)	Gashmard et al. (2020) [40]	Y	Y	Y	Y	Y	Y	Y	Y	Y	Y	A
(A5)	Lam and Mackenzie (2002) [41]	Y	Y	Y	Y	Y	Y	N	Y	Y	Y	A
(A6)	Deakin and Jahoda (2020) [42]	Y	Y	Y	Y	Y	Y	Y	Y	Y	Y	A
(A7)	Brantley et al. (2023) [43]	Y	Y	Y	Y	Y	Y	Y	Y	Y	Y	A
(A8)	Huiracocha et al. (2017) [44]	Y	Y	Y	Y	Y	U	N	Y	U	Y	B

Evaluation result: “Y”: Yes; “N”: No; “U”: unclear; “N/A”: Not applicable. JBI’s Critical Assessment Checklist for Qualitative Research: Q1: Are Philosophical Foundations and Methodology Consistent? Q2: Is the research objective or question consistent with the method used? Q3: Is the data collection method consistent with the method? Q4: Is the representation and analysis of data consistent with the method? Q5: Is the explanation consistent with the method used? Q6: Is there a statement regarding the cultural or theoretical positioning of researchers? Q7: Did the researchers influence the research, and vice versa? Q8: Are participants and their voices sufficiently representative? Q9: According to current standards, is the research ethical, or, in the case of recent research, is there evidence to suggest that ethical approval from appropriate institutions? Q10: Do the conclusions drawn from the research report come from the analysis or interpretation of the data?

### Data extraction and synthesis

To extract and evaluate data quality, standardised collection tables, including the year of publication, author, country, region, research methods, research subjects, scenarios, phenomena of interest, and research results, were used. The data were synthesised using a three-stage method [45] with two reviewers (ZXN and ZS) working together. In the first stage, all text fragments related to the experience and needs of caregivers of children with DS were encoded using the letter A and a number to encode and group the results represented in all tables. The encoding and grouping of the research results were guided by Smith’s interpretive phenomenological analysis [46]. The two reviewers repeatedly read the studies until no new codes were generated. In the second stage, the auditor generated a code list by categorising and grouping existing codes. Any differences between the reviewers were resolved by consulting the original text and the research team. In the third stage, descriptive themes and double checks were conducted to ensure accuracy and compliance. The data were revisited several times and revised until a consensus was reached among the reviewers on the identified codes and topics.

### Ethical consideration

As this study was a meta-synthesis, the requirement for ethical approval was waived.

## Results

### Search outcome

**Fig. 1:**
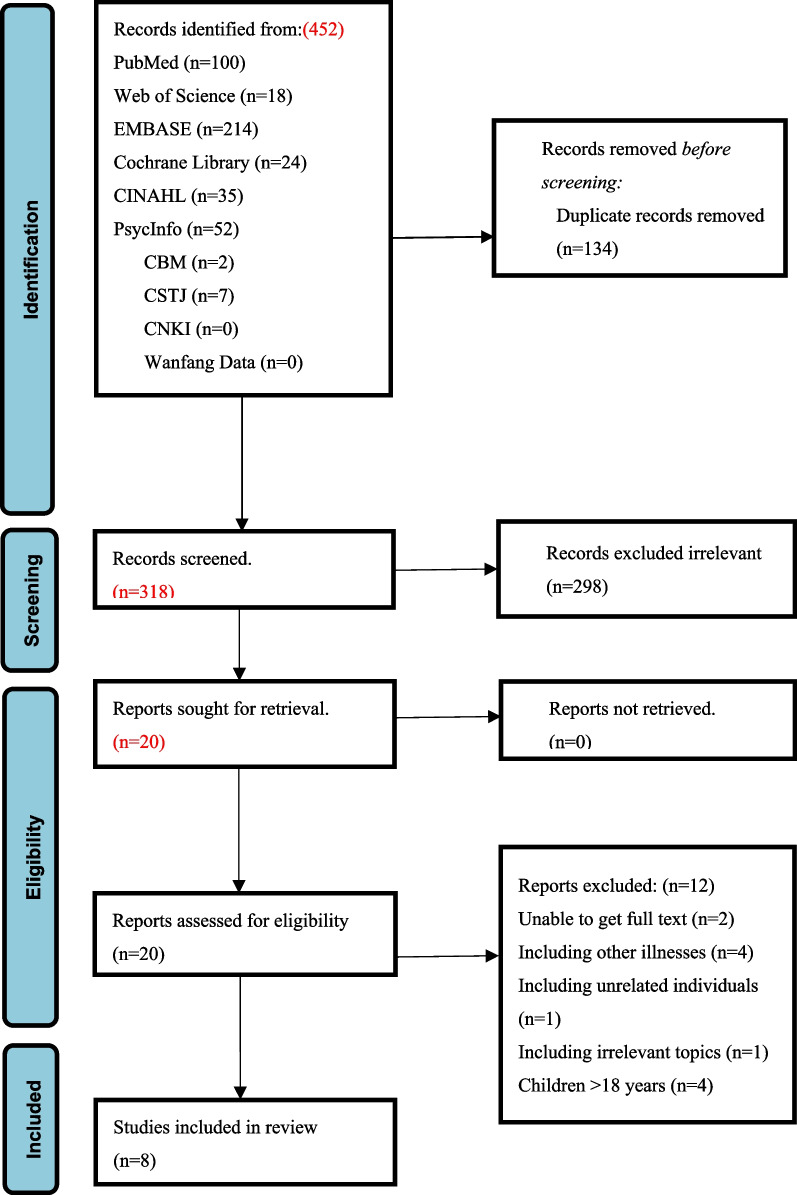
Flow chart

In total, 452 articles were retrieved through the preliminary search. After excluding duplicate studies (*n* = 134) using EndNote X9, 318 articles were included in the meta-synthesis. Screening was independently conducted by the first reviewer (ZXN), while the second reviewer (ZS) confirmed the screening by reviewing all abstracts and full texts and deleting additional studies based on titles and abstracts (*n* = 298). The remaining 20 studies were selected for full-text review and reference tracking, of which two studies were excluded due to inability to access the full text; four studies were excluded because they included children with other diseases; one study was excluded due to the inclusion of unrelated populations, such as healthcare workers; one study was excluded because it focused on the quality of life of parents of children with DS; and four studies were excluded due to the age of children with DS being > 18 years. Finally, eight articles published between 2002 and 2023 were included, and their reference lists were searched to identify new studies that met the inclusion criteria; however, none were found. The excluded articles were reviewed by a second reviewer (ZS), and any differences were resolved through discussion and consensus. The literature screening process is illustrated in Fig. 1.

### Study characteristics

Table 2 summarises the eight features included in this study [37–44]. The eight studies included 128 participants with different sociodemographic backgrounds, all of whom were caregivers of children with DS. The age range of the children was 0–18 years. These studies were conducted in both developed and developing countries, including China [37, 41], Türkiye [38], Britain [39, 42], Iran [40], America [43], and Ecuador [44]. All studies aimed to explore the care experiences and coping strategies of caregivers of children with DS. Among them, one study specifically reported on stigma experiences and perceptions of mothers of children with DS [42], while another specifically reported on the dietary and feeding pressures of children with DS [43]. After quality evaluation, eight articles were included in the study. Through repeated reading and analysis of the included articles, seven comprehensive findings were extracted: feeding pressure, hope for education, societal rejection and stigma, psychological pressure, care burden, family burden, family adaptation, and self-growth. Table 3 presents an overview of these findings.

**Table 2:** Summary table of data extraction

Author (year) county and article codes	Participants	Children age	Research method/ Sampling method	Scene	Phenomena of interest	Key findings
Zhang (2017) [37] China (A1)	*N* = 8 mothers(*n* = 2) grandmothers (*n* = 4) grandfather (*n* = 1) maternal grandmothers (*n* = 1)	2.5–8 years	Unstructured interview; Phenomenology/ Purposeful sample	Unknown	Psychological experience of main caregivers of DS children	1. Pain and helplessness 2. Under pressure, both physically and mentally exhausted 3. Feeling hopeless 4. Support and understanding
Çelik and Uzun (2023) [38] Turkey (A2)	*N* = 26 mothers (*n* = 23) fathers (*n* = 3)	0.5–4 years	Semi-structured interview/ Purposeful sample	Hospital	Stress experience of parents of DS children.	1. Emotional burden 2. Taking care of the burden 3. Combat stigmatization and discrimination 4. Worry about the future 5. Health and education challenges 6. Economic difficulties
Ridding and Williams (2019) [39] Britain (A3)	*N* = 13 fathers (*n* = 13)	0.5–8 years	Semi-structured interview; Grounded theory/ Purposeful sample	Home	The parenting adaptation of DS children’s fathers	1. Adapting to children 2. Adapt to the role of parents/spouses 3. Adapting to society
Gashmard et al. (2020) [40] Iran (A4)	*N* = 20 mothers (*n* = 10) fathers (*n* = 6) brothers (*n* = 2) sisters (*n* = 2)	4.5–18 years	Semi-structured interview/ Purposeful sample	Rehabilitation center, home	The coping strategies of families with DS patients	1. searching for information 2. Pay attention to the health needs of children 3. Focusing on faith 4. Teaching social skills 5. Enhancing self-reliance 6. Developing family support circles
Lam and Mackenzie (2002) [41] China (A5)	*N* = 18 mothers (*n* = 18)	2–6 years	Semi-structured interview; Qualitative research/ purposeful sample	Unknown	Experience of Chinese mothers raising children with DS	1. Unexpected birth of children with DS 2. Accepting the child 3. Special needs 4. Worry about the future 5. Lack of knowledge 6. Affects marital relationships 7. Social restrictions
Deakin and Jahoda (2020) [42] Britain (A6)	*N* = 9 mothers (*n* = 9)	9–16 years	Semi-structured interview; Phenomenology/ purposeful sampling	Home	The cognitive development of mothers towards their children’s disabilities and views on social stigma	1, Downplaying the significance of DS 2. Noticing stigmatized treatment 3. Looking for opportunities to inform the facts 4. Strive to treat all children equally 5. Gradually realizing one’s own illness
Brantley et al.(2023) [43] America (A7)	*N* = 15 mothers (*n* = 13) grandmothers (*n* = 2)	2–6 years	Semi-structured interview; Individual interview/ purposeful sampling; snowball sampling	Unknown	The sources of feeding pressure, resources, and coping strategies for caregivers	1. Get support from professionals 2. Undertake economic pressure 3. Change feeding situation or feeding issues 4. Emotional coping strategies
Huiracocha et al.(2017) [44] Ecuador (A8)	*N* = 19 mothers (*n* = 10) fathers (*n* = 9)	2–16 years	Focus group interview/ purposeful sampling	DS children’s specialized center	The impact of DS diagnosis on families	1. Inappropriate communication among medical personnel 2. Undertake additional work and responsibilities 3. Undertake economic pressure 4. Social discrimination 5. Father’s Escape

**Table 3:** Coding and grouping table

Research findings	Sub-themes	Synthesized themes
Difficulty swallowing (A2) Using assistive tools (A7) Delayed self-feeding (A7)	Feeding difficulty	1. Feeding pressure
Picky eating behavior (A7) Fear of new foods (A7)	Concerns about eating adequacy	
Positive coping (A7) Negative coping (A7)	Emotional-based coping	
School learning opportunities (A1) Peer relationship opportunities (A1) Having professional teachers (A2)	Equal access to education	2. Hope of education
Language communication disorders (A8) Early Language intervention (A8)	Early language development	
Discrimination against friends and relatives (A2) Discrimination against medical personnel(A2) Discrimination against family members (A5)	Discrimination and isolation in social interaction	3. Societal rejection and stigma
Boredom and rejection (A6) Avoidance behavior (A6)	Psychological barriers caused by discrimination and stigmatization	
Pain and helplessness (A1) Self doubt (A2) Shock and denial (A5)	Strong emotional fluctuations	4. Psychological pressure
A strong sense of uncertainty (A1) Entrust to potential caregivers (A2)	Worried about future belonging	
Consume strength (A1) Reduce rest time (A2) Give up work (A2) Abandon interests and hobbies (A8)	Chaotic work and life	5. Caring burden
Interpersonal tension (A3)	Social restrictions	
Support each other (A2) Self adaptation process (A3) Refusal to share responsibility (A5)	Affected marital relationships	6. Family burden
Stereotypes of gender (A3) Stereotypical male behavior patterns (A8)	Imbalanced gender roles	
Low family economic level (A2) High medical demand (A4)	Unbearable economic pressure	
Lack of professional knowledge (A1) Difficulty in obtaining information (A2) High demand for healthcare information (A4)	Knowledge deficit	
Positive and optimistic (A2) Remove prejudice (A2) Concentration on spirituality (A4)	Positive thinking and maintaining hope	7. Family adaptation and self-growth
Family support (A2) Professional support (A3) Social support (A7)	Search for information and obtain support	

### Themes and subthemes

Eight articles were included, and 43 research results were obtained. The research results were divided into 17 subthemes and seven comprehensive themes, as shown in Table 3. The synthesised themes were as follows: (1) feeding pressure, (2) hope for education, (3) societal rejection and stigma (4) psychological pressure, and (5) caring burden, (6) family burden, and (7) family adaptation and self-growth.

#### Theme 1: Feeding pressure

Children with DS have low muscle tone, weak oral muscles, and delayed development of the oral motor skills required for sucking, drinking, and chewing, which may cause difficulty in eating. Caregivers must spend additional time and energy helping them complete their eating processes. Children with DS have a limited availability of acceptable types of food, low dietary quality, and insufficient comprehensive nutrient intake, inducing varying degrees of psychological pressure on caregivers.

### Feeding difficulty

Children with DS have difficulty chewing and swallowing and are prone to coughing or aspiration. Therefore, it is necessary to change the texture of food and supervise eating. Children need to use special straws, bottles, and cups to assist in the feeding process and improve their self-feeding abilities. Caregivers experience additional psychological pressure due to concerns about their child’s coughing, aspiration, and the inability to communicate with others after coughing. They attempt to utilise various professional and interpersonal resources and consult nurses and various therapists (such as occupational, physical, and language therapists) to obtain support for skill development and optimise nutrition.

‘I had difficulty feeding her. She spits. She was unable to swallow solid food. I must feed her while playing. This takes up a lot of time.’ [38].

‘We have every spoon and every kind of utensil. She has Chew Tubes…Z Vibe.’ [43].

‘Her occupational therapist that would come out here when she was in early intervention was one of the best sources for me…’ [43].

### Concerns about eating adequacy

Several caregivers reported stress and fear regarding new foods because of the limited availability of acceptable foods. The picky eating behaviour of some children with DS leads to a decline in dietary quality. Caregivers obtain information by consulting professionals. They try to cope with picky eating behaviours by mixing multiple foods into smoothies, preparing or cooking food in advance, and playing music to distract their attention during meals. Feeding difficulties affect the physical and emotional life of children with DS. Sucking, insufficient chewing, prolonged eating time, and large emotional fluctuations over time can lead to a significant increase in the incidence of oral movement problems, weight loss, or obesity.

‘He doesn’t like vegetables. He doesn’t like any fruits besides bananas.’ [43].

‘There was a time where things were so bad with him not drinking from a cup and that was really stressful.’ [43].

‘Music. So, if we play some music, or if I put on like Coco Melon or Elmo on my phone and like prop it up against the basket, and then set her in the chair, then it’s like magic she eats.’ [43].

### Emotional-based coping

The family economic status and resource support may become sources of feeding pressure for caregivers. High economic levels and sufficient professional resources can increase feeding confidence. Caregivers often exhibit anxiety and stress when experiencing feeding difficulties. Coping strategies based on this negative emotion include positive coping, such as Mindfulness-Based Stress Reduction (MBSR), meditation, yoga, etc., as well as negative coping, such as a stress-related diet. MBSR has been found to be very effective in reducing pressure on caregivers of children with disabilities and chronic diseases.

‘Getting out on a walk or long showers help me a lot. Just taking me time by myself without the kids around.’ [43].

‘I am off on Thursdays and Fridays, and I will sit and eat chips. I think that’s my main thing. Chips and chocolate…if I’m home, then I’ll just sit and eat and watch TV. Like I’ll have chips after breakfast. And I’ll enjoy it. I don’t ever think of like, good and bad. It does make me feel bad sometimes, but it’s okay.’ [43].

‘We were going to an occupational therapist that specialised in nutrition before the pandemic started. And she would encourage me just to have him be present around food.’ [43].

#### Theme 2. Hope of education

Children with DS show limited development in intelligence, language, behaviour, and other aspects. Regular primary schools are usually unable to accept their participation in formal academic subjects (including mathematics and English), and only a few schools allow them to be included in mainstream classrooms during mealtimes, music classes, and social activities. They are also usually unable to enter secondary school to continue their education [47]. Caregivers urgently hope that their children have equal educational opportunities; however, most caregivers take care of children with DS for the first time and lack basic knowledge of the disease, language intervention, rehabilitation treatment, and other related professional knowledge reserves. They hope to acquire professional knowledge and skills through the guidance of professionals, integrate treatment into daily life, and promote improved recovery and growth of children.

### Equal access to education

Most regular schools lack experience in caring for children with disabilities, are unwilling to spend extra time on this task, and refuse to accept such children for enrolment. Some special education schools hire physical, occupational, and language therapists to learn sign language with children, help them communicate better, and engage in professional exercises to improve low muscle tone. However, not all special education schools provide comprehensive services and the quality of care within schools varies. Caregivers are concerned that their children may not receive good services [43], resulting in many caregivers postponing the initiation of education for children with DS. As people age, the gap between children with DS and their peers gradually becomes apparent, and caregivers urgently hope that their children can enter school and receive an equal education.

‘However, they found several kindergartens that did not charge extra childcare fees. This is because they do not have the time to take care of such children.’ [37].

‘Whenever other children play, my grandson silently watches beside them. Although he does not know how to play, I feel like he is willing to watch, hoping he has the opportunity to play and learn like a healthy child.’ [37].

‘I think the child development specialist doesn’t have enough experience, so I have concerns. They do not let us enter the educational room (with our children). I do not know what has been done (during the special education sessions).’ [38].

‘They experience high levels of exhaustion during the school process. They are blamed for their disability at school…No one gives support.’ [38].

### Early language development

Children with DS have a certain degree of developmental delay and learning disabilities [48], and most developmental delays are related to language and communication. This is mainly related to children’s speech disorders and hearing and comprehension problems, accompanied by learning delays and difficulties in learning new things. Caregivers crave broader help and resources to provide early and ongoing language intervention for children with DS and incorporate language intervention into daily family activities such as eating, bathing, dressing, and playing [49].

‘How long it takes to learn something new and how challenging it can be even for the simplest task.’ [49].

‘Progress comes slowly. He feels as though he is on the verge of his next milestone, but it may take 3 months to get him over the edge.’ [49].

#### Theme 3. Societal rejection and stigma

The acceptance of children with congenital diseases in society is relatively low, and children with DS often suffer rejection and stigmatisation from the community, neighbours, health professionals, relatives, family members, and strangers during their daily social interactions. Long-term negative attitudes and differential treatment cause children with DS to experience anxiety and attempt to adopt avoidance strategies, exerting enormous psychological pressure on children with DS and their caregivers.

### Discrimination and isolation in social interaction

Despite inclusive policies adopted by many countries, the insults and exclusions faced by children with DS and their families persist. Many people refuse to have their children play with children with DS and even suspect that DS is contagious. Parents attempt to prevent social isolation and expand correct and safe social interactions with their children by helping them develop social interactions with healthy peers. However, the effectiveness of these measures is limited.

‘She (my friend) didn’t know whether the syndrome was infectious or not…While her son was together with my son, people stared at both of them…In addition, she was afraid that her son would imitate my son’s behaviour. Therefore, she no longer came into contact with me.’ [41].

‘Some of our relatives look at my child as if (he/she) has a contagious disease and do not allow them to play with (him/her).’ [40].

‘I have taught my child how to greet and introduce oneself to others and how to ask peers to play games with.’ [40].

‘The comments were not only from strangers but also from those closest to me, including my mother-in-law.’ [38].

‘They called me ‘the mother of the boy with Down’… I had never heard of it before.’ [38].

### Psychological barriers caused by discrimination and stigmatisation

The gazes of local residents and stigmatising behaviours focused on special appearances or intellectual disabilities that caused rejection and annoyance among children with DS. Long-term negative treatment exerts enormous psychological pressure on children with DS, leading to feelings of inferiority and avoidance. Parents have attempted to reduce the impact of discrimination and stigma on children with DS by concealing their medical conditions; however, these measures have been limited.

‘If someone is staring at her and she starts to feel uncomfortable, say we’re in McDonald’s or a restaurant or whatever, it’s just she’ll start that, you know the rocking, that’s her getting agitated.’ [42].

‘Because they call them little imbeciles (tontitos).because people keep staring at them.because they are made fun of.or because people are tired of them.’ [44].

#### Theme 4: Psychological pressure

Families of children with DS must accept within a short period that their children are born with disabilities, which causes them to experience strong emotional fluctuations. Children with DS have poor self-care abilities, delayed intellectual development, and subsequent treatment and education, and the future belonging of the child brings enormous psychological pressure on caregivers.

### Strong emotional fluctuations

DS caregivers are initially shocked when they learn that their children are sick and usually try to deny this fact. DS severely affects daily life and cannot be cured, causing pain and helplessness in caregivers. Caregivers are concerned about their children’s health and education and how to raise them in the future. Caregivers worry about who will take care of their children when they are not present.

‘I am very emotional…Will I be enough for my child? Will I be able to meet his needs? Will I be able to offer him a good education?’ [38].

‘I was scared of him. I thought he might be a terrible creature. I tried to resist touching him. I did not believe that he was my own baby…I did not dare look at him and I did not want people to ask him. I cried when I thought of him or if nobody mentioned him.’ [42].

### Worried about future belonging

Caregivers often exhibit a strong sense of uncertainty regarding the affected child’s future belonging. Caregivers are generally worried about the ability of children with DS to make a living and become independent after they leave. They attempt to provide lifelong support to children with DS by asking for another child or allowing older brothers and sisters to act as potential caregivers.

‘I do not know what my child will be like in the future. I cannot spend my entire life with him. Who will take care of him when he ages? I cannot imagine what his future will be like.’ [37].

‘He has a 4-year-old sister, but her younger sister doesn’t like him very much now. Who can take care of them all the times when we are gone?’ [37].

‘I am considering having another child, just for her sake, to always be by her side…At least, there may be a sibling at home who supports her.’ [38].

#### Theme 5: Caring burden

Children with DS are unable to care for themselves and require dedicated caregivers, which disrupts the caregiver’s original work and life, increases their physical exertion, and may even sacrifice their original work. Repetitive daily care tasks narrow the caregivers’ social scope and cause tension in their social relationships.

### Chaotic work and life

Children with DS lack the ability to care for themselves and rely on caregivers in their daily lives. Caregivers face high-intensity caregiving roles and responsibilities throughout the day. Caregivers must invest extra time in supporting their children’s development and learning, which forces them to give up their original jobs or sacrifice their interests and hobbies.

‘Taking care of him is really difficult. You have to keep watching him step-by-step, thinking about everything for him. If you do not pay attention, he will fall.’ [37].

‘I am a chess artist. I used to play chess frequently when I had free time, but now I cannot spare time.’ [37].

‘I gave up going to the gym to which I had gone every morning. I stopped visiting my parents.I stopped going out.I began to live each day alone with my daughter and I began to focus on doing all the practical things that I could.’ [44].

### Social restrictions

Caregivers must accompany their children for training, conduct training at home, and take them to subsequent appointments and evaluations. Spending so much energy exhausted the caregivers; they lacked time to socialise, and their interpersonal relationships tended to be tense. Most brothers and sisters of children with DS are willing to accept them and take care of them, which is conducive to family unity. Some younger children sometimes envy their parents’ extra attention to children with DS, and some older children do not tell their friends and classmates that they have a brother or sister with DS [41]. Mothers are concerned that their children with DS may experience adverse effects on the social interactions of other children.

‘Just getting away for a night…you can’t leave Jay alone. In 6.5 years, we have spent two nights away from Jay and that was not far away.’ [39].

‘I always explain my child’s physical and mental problems to my other children and remind them that he needs extra care, and they also help as they can.’ [40].

#### Theme 6: Family burden

As the primary caregivers of children with DS, parents jointly bear the obligation of upbringing. Marital relationships are adversely affected when family responsibilities are unevenly distributed within the family or when one spouse is unwilling to take care of children with DS. Fathers play a crucial role in marital relationships as the main supporters of mothers. Raising children with DS requires expensive medication, treatment, and rehabilitation training, which place an economic burden on families. Most families care for children with DS for the first time and lack professional knowledge. They hope to acquire more professional knowledge and skills through the guidance of professionals to provide better care for their children.

### Affected marital relationships

Parents of children with DS can provide emotional support to each other, actively share the task of caring for their children and daily household chores, and overcome difficulties and challenges together, which has a positive effect on marital relationships. Some spouses show indifference towards their children and refuse to share the responsibility of taking care of them, which has a negative impact on marital relationships. When the father begins to accept and participate in the process of caring for the child, the mother’s burden is reduced, and the marital relationship improves.

‘Our marital relationship has strengthened. They supported each other. My husband takes cares of my boy. He told me that this was God’s decree. He consoled me and stated that this was not an issue related to the mother’s age. My greatest supporter is my husband.’ [38].

‘During that period, we had lots of arguments…He played mahjongg every night after work…He did not know how much time, effort, and patience I had spent with the child. I spoke to him and asked him to share some care responsibilities. However, he felt that I was complaining and that it was meaningless to continue the discussion.’ [41].

### Imbalanced gender roles

Influenced by stereotypes, mothers are seen as spiritual hubs of the family responsible for caring for children and household chores, whereas fathers mainly provide financial support to the family. Most mothers blame themselves and bear high psychological pressures because their children have disabilities. Although many fathers of children with DS have realised that taking care of their children is the joint responsibility of spouses, mothers are more involved in their child’s interventions and parenting practices than fathers.

‘The stereotype of the female carer (still exists)…a lot of the dads step back.’ [39].

‘I took for granted that my husband worked; he has a job.all the others are busy… In effect, it was me who more or less took on all of the care.because he is my son.’ [44].

### Unbearable economic pressure

Children with DS have high medical demands and require additional care, regular training, and frequent hospital visits, which increase family expenses. Many families have lower socioeconomic levels and caregivers give up work to take care of their children, resulting in reduced family income and difficulty in paying high medical expenses.

‘They could hardly afford their basic needs, pay rent, or transport to the hospital with a home care allowance.’ [38].

‘And so sometimes it’s stressful, like financials, financially, things are stressful…We definitely work within a budget, you know, because my husband is working, as opposed to if we both were working.’ [43].

‘We would have to pay a taxi to go and a taxi to come back, to buy medicines for my daughter, milk.sometimes it was not possible to arrange therapy at the social security and when we had a bit of money we’d pay for private therapy.but we could not always.sometimes there just was no money for it.’ [44].

### Knowledge deficit

Although DS cannot be cured, caregivers hope to improve their child’s symptoms through their own efforts. They persistently sought professional help, understood the characteristics and needs of children with DS, learned sensitive communication skills, and focused on family centred care. Due to the uneven distribution of existing medical resources, incomplete support services, and limited access to knowledge, caregivers hope to seek more convenient ways of acquiring professional knowledge.

‘I know this disease cannot be cured, but is there any way to make the child better? As long as it works, I still need to learn it in my 60s.’ [37].

‘I do not always believe my grandson does this. My daughter’s friend is a doctor and she says that regular training can improve things, but I do not know how to train.’ [37].

#### Theme 7: Family adaptation and self-growth

The birth of children with DS causes an initial physical and mental burden on caregivers. Internal support from family members and external support from social professionals effectively alleviated pressure on caregivers. They gained positive experiences in their daily interactions with their child, discovered new perspectives in life, ultimately gradually adjusted their mindsets, eliminated existing biases, and held optimistic hopes for children with DS.

### Positive thinking and maintaining hope

Caregivers express optimistic thoughts and hopes towards their children, have positive experiences in interacting with them, realise the importance of life, and believe that children have the potential to overcome their difficulties. Raising children with DS has changed caregivers’ perspectives on the world, themselves, and others, eliminating prejudices against the appearance of children with DS, other disabled children, and families of disabled children. They strive to promote and support the development of their children by setting up separate rooms for physical therapy at home, saving money for their children’s future [38], and sharing care responsibilities [39].

‘He is our baby. We said, “We wanted him.” We did not see him differently…He is a very pleasant child with a smiling face and a big, loving heart…He smiles with his eyes. I have raised another child, but he is unique.’ [38].

‘After her birth, I have learned not to judge people by their appearance. I have turned off this bias. All things could be settled with talk and love.’ [38].

‘This child is pure and innocent. When I spend time with him, I have the impression that one of God’s angels is with me and whenever I do something for him, I have a spiritual feeling. I feel pleased.’ [40].

### Search for information and obtaining support

In terms of family support, the most supportive family members were spouses, and higher levels of spousal support and marital quality were related to better coping with challenging life events or negative environments [50]. The attitudes of society towards children with DS have undergone positive changes, and most caregivers have expressed full support from society. Social support is an important resource for parents of children with DS for managing stress and acceptance [51, 52]. To gain a more comprehensive understanding of the disease and provide better treatment for children, caregivers persistently seek help from the child’s counsellors, professional medical staff in rehabilitation and vocational training centres, and language therapists.

‘This situation brought fathers comfort. Grandfathers, grandmothers, and other family members supported the father not only mentally but also in daily life.’ [40].

‘During pregnancy, we did not know what Down syndrome was. We started researching and joined a ‘WhatsApp’ family group…I continued to wonder, research, and follow family groups.’ [38].

‘I feel fortunate that we’ve had her now and not 20 years ago ‘cos it just seems a lot’s happened in… 20 years.’ [39].

‘Twice a week, I take him for occupational and speech therapy. During the occupational therapy, she played sports and painted. She greatly improved following such therapies.’ [40].

## Discussion

This meta-synthesis explored the experiences of caregivers in raising children with DS as well as changes in their psychological experiences, resulting in different themes. After summarising and organising the literature, seven prominent themes emerged (feeding pressure, hope for education, societal rejection and stigma, psychological pressure, care burden, family burden, family adaptation, and self-growth.). There are differences in the structure and function of the mouth and throat in children with DS, which affect eating and swallowing [53], and mainly manifest as poor chewing, difficulty in swallowing, vomiting, aspiration, and suffocation [54]. In all included studies, feeding stress in children with DS was an important source of stress for caregivers, consuming a significant amount of time and energy [38, 41, 43]. Caregivers attempt various measures, such as changing the texture of food and using assistive devices, to help children eat better [43]. Previous studies have shown that thickened drinks improve aspiration and swallowing difficulties in children with DS [55]. Interventional therapies aimed at cultivating oral motor skills and improving muscle tone promote oral development and eating independence in children with DS [56]. In this regard, registered nutritionists with work experience with children can be advocated to provide medical nutrition treatment and manage diet-related health issues in children with DS. Nurses can assist caregivers in observing their daily diet to ensure food safety.

This meta-synthesis revealed that caregivers’ hopes for education included the desire for their children to have equal educational opportunities and the development of early language functions. Current mainstream and special education schools have inadequate educational services for children with special needs, and the educational needs of many children with DS cannot be met [37, 38]. Schools should enhance the professionalism of their staff, increase inclusivity towards children with special needs, strengthen communication with their parents, and enable them to fully understand the support their children receive [57, 58]. Although children with DS have special educational needs, they can thrive academically and socially in mainstream education through inclusive placement (students earning 80% or more credit in a regular educational environment) and positive interactions between teachers and students [59, 60]. Schools or kindergartens provide long-term patient education and training for children, preparatory education for children to transition to regular schools, and encourage children to play and engage in activities with healthy children, which is of great help to their growth and can greatly reduce the pressure on caregivers.

Active parental involvement in children’s language interventions may help alleviate challenges related to parent-child communication [61]. Mothers of children with DS typically use more language than do fathers and children [62]. Fathers’ cognitive and verbal stimuli to infants with developmental disorders are positively correlated with children’s cognitive and language development [63]. Providing training to parents about the importance of responsivity and the quality and quantity of their language input and interaction encourage active participation in language interventions for children with DS. Guiding parents to use positive interactive methods in their daily interactions with children with DS; ensuring diverse language input; and maximising the development of children’s cognition, social interaction, and communication skills encourage effective parental involvement [64].

Many caregivers experience discrimination and stigmatisation from friends, relatives, and medical personnel while raising children with DS [38, 40–42, 44]. Most parents tend to use negative coping strategies, including concealing their child’s diagnosis, not disclosing information about their child, avoiding social contact, and ignoring comments or behaviours that belittle their child [65]. This had a significant negative impact on parents’ sense of happiness, self-esteem, and self-efficacy. Children with DS can become aware of negative stereotypes related to DS, which may negatively impact their mental health [66]. Hospitals should strengthen the training of sensitive communication skills for medical personnel, and nursing staff should educate the public that DS is not contagious and should not be feared or despised in order to assist children in integrating themselves into society. Society can increase public awareness of DS and reduce bias through regular online knowledge pushes and offline public welfare promotional activities.

Among the eight included studies, a large proportion of caregivers reported experiencing varying degrees of psychological stress [37, 38, 41, 44], consistent with previous studies [67]. These pressures arise mainly from daily care burdens [37, 38, 41, 44], external discrimination [37, 38, 44], family economic conditions [38, 44], differences between children and their normal peers [37], and concerns about the future [38]. All caregivers mentioned the heavy burden of daily care, which may be a more common stressor than other stressors. Most mothers experience greater psychological pressure than other caregivers of children with DS [38, 42, 44]. They blame themselves for their children’s disabilities [38], believe they are cursed, have a blood relationship with their husbands, are older, do not undergo regular checkups during pregnancy, or unintentionally do something wrong [38]. Female caregivers experience higher levels of burden, tension, anxiety, and depression than male caregivers [68], which may be because mothers play the primary caregiver role in the family, making them more susceptible to physical, emotional, and economic burdens as well as restrictions on leisure and social activities [69]. Institutions, such as societies and hospitals, should pay attention to the psychological pressure faced by caregivers of children with DS, especially mothers, and provide psychological counselling when necessary to help them overcome difficulties more quickly.

Caregivers spend a lot of time taking care of the daily lives of children with DS, compressing their rest, social, and entertainment time [37, 39, 44] and even leading to interpersonal tension [39]. This is consistent with the survey results of caregivers of children with disabilities [67], who often give up their social relationships and need to care for their families. Long-term care responsibilities lead to poor mental and physical health in caregivers [70]. Current nursing services do not address caregivers’ health and wellbeing issues, and the care burden borne by caregivers is often underestimated and lacks sufficient support [71]. This should be widely recognised by the society and healthcare workers.

A meta-synthesis found that the family burden may depend on marital relationships and family economic situation. The birth of children with DS has a negative impact on the marital relationships of some families, which was most significant in a study on families in Hong Kong [41]. This may be related to the traditional social beliefs in the region that having disabled children is a ‘disgraceful family matter.’ This sense of shame may lead parents to reduce taking their children to public places to avoid unfriendly looks from the outside world, resulting in a gradual decline in their self-confidence and social participation. Some fathers could not accept their children, reduced their care for them, or stayed away from their families. Thus, it increases the burden on the mother’s care and reduces the quality of marriage. Pain experienced by parents of children with developmental disabilities is negatively correlated with marital quality [72]. The average divorce rate of parents with disabled children is 6% higher than that of parents without disabled children [73]. In this regard, grandparents or nannies can be advocated for providing temporary care to children with DS. Temporary care can provide time for parents of children with DS to participate in promotional activities, reduce couples’stress levels, and improve the quality of marriage.

The family’s economic situation is closely related to family burden, and most caregivers indicate that children with DS need regular treatment and rehabilitation training [37, 38, 41], A study in Changsha, China showed that the average life-cycle economic burden of patients is 4.9857 million yuan [74]. Parents attempt to meet their children’s medical and educational needs by reducing their daily expenses, which results in a decline in the quality of family life [38, 44]. Some caregivers have improved their ability to take care of disabled children by reducing working hours or resigning, but their career opportunities and income levels have decreased, and the economic burden on families has further increased [75].

The primary way for families with DS to acquire parenting knowledge and skills is through medical and nursing teams [40]. Due to the lack of medical resources in certain regions, to expand information sources, they choose to search the Internet and actively join social organisations related to DS for mutual communication and learning [38–40]. Families of children with DS urgently need to participate in educational seminars and thematic discussions related to DS, read educational brochures and books, and talk with their peers. Hospitals should consider opening telemedicine and online consultation services to provide online guidance for families of children with DS, helping them obtain necessary medical advice and support. Local hospitals should be encouraged to establish partnerships with social organisations related to DS, provide education and advice on the importance of maintaining health, regularly organise lectures, and provide platforms for communication and learning for families with DS.

Family adaptation refers to a family’s efforts to bring balance, harmony, and functionality into a crisis [76]. Successful adaptation occurs when families strike a balance between the needs of children with DS, other family members, and the entire family [77]. Most caregivers gradually achieve family adaptation and self-growth after experiencing a series of internal and external challenges after the birth of children [38–41]. Adaptation methods include changing one’s pessimistic attitudes towards children with DS and actively seeking support from the outside world [38–40]. Simultaneously, caregivers become more compassionate and inclusive, thereby achieving self-growth. Although the birth of children with DS adversely affects families, communities, and society, increasing evidence suggests that many families have successfully adapted to the challenges associated with raising children with DS [78, 79]. People have found that family communication skills, cohesion among members, and the support and quality of health services contribute to family adaptation [6]. Therefore, DS public welfare organisations and hospitals can provide targeted social support and medical assistance to families of children with DS, helping them complete the adaptation process faster.

### Advantages and limitations

Presently, most research focuses on the caregiver experiences for children with developmental/intellectual disabilities and we focused on articles on the caregiver experiences for children with DS. DS has received considerable attention in recent years and focusing on a particular disease can make research results more targeted. Healthcare providers should guide caregivers by providing advice and support in meeting their children’s unique needs. The results of this review were carefully interpreted considering their potential limitations. This review included only qualitative research published in both English and Chinese, and grey literature may be overlooked. This might have led to the omission of important evidence from other sources.

### Future research

Most studies included in this review focused only on the parents of children with DS. Other immediate family members, such as grandparents of children with DS, also participate in their daily care, and culture, age, and role differences among these caregivers can have different effects on their care experiences. It would be meaningful to explore the unique experiences of different caregivers through independent research or interviews with grandparents, brothers, sisters, and other immediate family members who participate in a child’s daily care.

Future research should delve deeper into the effects of cultural and racial diversity among caregivers. This may involve exploring the cultural beliefs and practices of different populations in different regions as well as how societal attitudes towards DS affect caregiver care.

Research could also focus on identifying factors that promote or hinder daily care, such as difficulties in accessing resources, social support, and caregiver health status, and developing targeted interventions to address these factors. This may reduce the physical and mental burden on caregivers and improve their quality of life and care experiences.

Finally, it would be beneficial to explore the effectiveness of various support mechanisms, such as caregiver training programmes, support groups, and respite care services, in helping caregivers manage their responsibilities and achieve a better quality of life. This could provide valuable information for healthcare professionals, policymakers, and DS public welfare organisations and provide timely, sufficient, and continuous support to families of children with DS.

## Conclusion

This study adopted a summary and integration method to conduct a meta-integration of qualitative research related to this theme, exploring the care burden, real emotional experiences, coping strategies, and self-growth of caregivers of children with DS. This meta-synthesis of findings indicates the necessity of establishing understanding and support for families of children with DS. The results of this comprehensive research will provide a reference for the future efforts of medical institutions, especially paediatric healthcare professionals, to formulate support policies, regularly provide psychological care for caregivers of children with DS, increase scientific popularisation efforts to raise public awareness, and provide timely, sufficient, and continuous support. This is crucial for enhancing caregivers’ adaptability and enabling them to achieve self-growth as soon as possible.

## Supplementary Information

Supplementary Material 1.

## Data Availability

All data generated or analysed in this study are included in the published article [and supplementary information files].

## Abbreviations

PRISMA: Preferred Reporting Items for Systematic Reviews and Meta-synthesis
CBM: China Biology Medicine
CNKI: China National Knowledge Infrastructure
CSTJ: China Science and Technology Journal Database
DS: Down syndrome
ADHD: Attention Deficit Hyperactivity Disorder
ASD: Autism Spectrum Disorder
CASP: Critical Assessment Skills Program
