# Supplementary material for: Caregivers’ experience of having a child with Down syndrome: a meta-synthesis
Source: BMC Nurs. 2025 Jan 20;24:66. doi: 10.1186/s12912-024-02652-y (PMC11744819; doi:10.1186/s12912-024-02652-y)
Supplement: Supplementary file 1 — Supplementary Material 1. [file 12912_2024_2652_MOESM1_ESM.docx]

Table S1. Search Strategy

| **Databases** | **Search No.** | **Query** | **Results** |
| --- | --- | --- | --- |
| **Pubmed (MeSH)** | #1 | Search: "Down Syndrome"[Mesh] Sort by: Most Recent | 26716 |
|  | #2 | Search: ((((((((((((Syndrome, Down[Title/Abstract]) OR (Mongolism[Title/Abstract])) OR (47,XY,+21[Title/Abstract])) OR (Trisomy G[Title/Abstract])) OR (47,XX,+21[Title/Abstract])) OR (Down's Syndrome[Title/Abstract])) OR (Downs Syndrome[Title/Abstract])) OR (Syndrome, Down's[Title/Abstract])) OR (Trisomy 21[Title/Abstract])) OR (Trisomy 21, Mitotic Nondisjunction[Title/Abstract])) OR (Down Syndrome, Partial Trisomy 21[Title/Abstract])) OR (Partial Trisomy 21 Down Syndrome[Title/Abstract])) OR (Trisomy 21, Meiotic Nondisjunction[Title/Abstract]) | 16282 |
|  | #3 | #1 OR #2 | 31246 |
|  | #4 | Search: "Caregivers"[Mesh] Sort by: Most Recent | 52103 |
|  | #5 | Search: ((((((((((((((((Caregiver[Title/Abstract]) OR (Carers[Title/Abstract])) OR (Carer[Title/Abstract])) OR (Care Givers[Title/Abstract])) OR (Care Giver[Title/Abstract])) OR (Spouse Caregivers[Title/Abstract])) OR (Caregiver, Spouse[Title/Abstract])) OR (Caregivers, Spouse[Title/Abstract])) OR (Spouse Caregiver[Title/Abstract])) OR (Family Caregivers[Title/Abstract])) OR (Caregiver, Family[Title/Abstract])) OR (Caregivers, Family[Title/Abstract])) OR (Family Caregiver[Title/Abstract])) OR (Informal Caregivers[Title/Abstract])) OR (Caregiver, Informal[Title/Abstract])) OR (Caregivers, Informal[Title/Abstract])) OR (Informal Caregiver[Title/Abstract]) | 71662 |
|  | #6 | #4 OR #5 | 92222 |
|  | #7 | interview*[Title/Abstract] OR interviews[MeSH:noexp] OR experience*[Text Word] OR qualitative[Title/Abstract] | 1945903 |
|  | #8 | #3 AND #6 AND #7 | 100 |
| **EMBASE (emtree)** | #1 | 'down syndrome'/exp OR 'down syndrome' | 44514 |
|  | #2 | 'syndrome, down':ab,ti OR 'mongolism':ab,ti OR '47,xy,+21':ab,ti OR 'trisomy g':ab,ti OR '47,xx,+21':ab,ti OR 'downs syndrome':ab,ti OR 'trisomy 21':ab,ti OR 'trisomy 21, mitotic nondisjunction':ab,ti OR 'down syndrome, partial trisomy 21':ab,ti OR 'partial trisomy 21 down syndrome':ab,ti OR 'trisomy 21, meiotic nondisjunction':ab,ti | 11247 |
|  | #3 | #1 OR #2 | 49839 |
|  | #4 | 'caregiver'/exp OR 'caregiver' | 142414 |
|  | #5 | 'caregiver':ab,ti OR 'carers':ab,ti OR 'carer':ab,ti OR 'care givers':ab,ti OR 'care giver':ab,ti OR 'spouse caregivers':ab,ti OR 'caregiver, spouse':ab,ti OR 'caregivers, spouse':ab,ti OR 'spouse caregiver':ab,ti OR 'family caregivers':ab,ti OR 'caregiver, family':ab,ti OR 'caregivers, family':ab,ti OR 'family caregiver':ab,ti OR 'informal caregivers':ab,ti OR 'caregiver, informal':ab,ti OR 'caregivers, informal':ab,ti OR 'informal caregiver':ab,ti | 100634 |
|  | #6 | #4 OR #5 | 164647 |
|  | #7 | 'interview':ab,ti OR 'interviews':ab,ti OR 'experience':ab,ti OR 'qualitative':ab,ti | 1900005 |
|  | #8 | #3 AND #6 AND #7 | 214 |
| **Cochrane Library** | #1 | MeSH descriptor: [Down Syndrome] explode all trees | 520 |
|  | #2 | (Syndrome, Down):ti,ab,kw OR (Mongolism):ti,ab,kw OR (Trisomy G):ti,ab,kw OR (Down's Syndrome):ti,ab,kw OR (Downs Syndrome):ti,ab,kw OR (Syndrome, Down's):ti,ab,kw OR (Trisomy 21):ti,ab,kw OR (Trisomy 21, Mitotic Nondisjunction):ti,ab,kw OR (Down Syndrome, Partial Trisomy 21):ti,ab,kw OR (Partial Trisomy 21 Down Syndrome):ti,ab,kw OR (Trisomy 21, Meiotic Nondisjunction):ti,ab,kw | 2447 |
|  | #3 | #1 OR #2 | 2447 |
|  | #4 | MeSH descriptor: [Caregivers] explode all trees | 3758 |
|  | #5 | (Caregiver):ti,ab,kw OR (Carers):ti,ab,kw OR (Carer):ti,ab,kw OR (Care Givers):ti,ab,kw OR (Care Giver):ti,ab,kw OR (Spouse Caregivers):ti,ab,kw OR (Caregiver, Spouse):ti,ab,kw OR (Caregivers, Spouse):ti,ab,kw OR (Spouse Caregiver):ti,ab,kw OR (Family Caregivers):ti,ab,kw OR (Caregiver, Family):ti,ab,kw OR (Caregivers, Family):ti,ab,kw OR (Family Caregiver):ti,ab,kw OR (Informal Caregivers):ti,ab,kw OR (Caregiver, Informal):ti,ab,kw OR (Caregivers, Informal):ti,ab,kw OR (Informal Caregiver):ti,ab,kw | 18495 |
|  | #6 | #4 OR #5 | 18968 |
|  | #7 | (interview):ti,ab,kw OR (interviews):ti,ab,kw OR (experience):ti,ab,kw OR (qualitative):ti,ab,kw | 115511 |
|  | #8 | #3 AND #6 AND #7 | 24 |
| **Web of Science** | #1 | TI=(Down Syndrome OR Syndrome, Down OR mongolian OR 47,XY,+21 OR Trisomy G OR 47,XX,+21 OR Down's Syndrome OR Downs Syndrome OR Syndrome, Down's OR Trisomy 21 OR Trisomy 21, Mitotic nondisjunction OR Down Syndrome, Partial Trisomy 21 OR Partial Trisomy 21 Down Syndrome OR Trisomy 21, Meiotic nondisjunction) | 25494 |
|  | #2 | TI=(Caregivers OR Caregiver OR Carers OR Carer OR Care Givers OR Care Giver OR Spouse Caregivers OR Caregiver, Spouse OR Caregivers, Spouse OR Spouse Caregiver OR Family Caregivers OR Caregiver, Family OR Caregivers, Family OR Family Caregiver OR Informal Caregivers OR Caregiver, Informal OR Caregivers, Informal OR Informal Caregiver) | 33983 |
|  | #3 | TS=(interview OR interviews OR experience OR qualitative) | 2306706 |
|  | #4 | #1 AND #2 AND #3 | 18 |
| **CINAHL** | #1 | (MH "Down Syndrome") | 8305 |
|  | #2 | TI Syndrome, Down OR mongolian OR 47,XY,+21 OR Trisomy G OR 47,XX,+21 OR Down's Syndrome OR Downs Syndrome OR Syndrome, Down's OR Trisomy 21 OR Trisomy 21, Mitotic nondisjunctive OR Down Syndrome, Partial Trisomy 21 OR Partial Trisomy 21 Down Syndrome OR Trisomy 21, Meiotic nondisjunctive | 6307 |
|  | #3 | #1 OR #2 | 9424 |
|  | #4 | (MH "Caregivers") | 44514 |
|  | #5 | TI Caregiver OR Carers OR Carer OR Care Givers OR Care Giver OR Spouse Caregivers OR Caregiver, Spouse OR Caregivers, Spouse OR Spouse Caregiver OR Family Caregivers OR Caregiver, Family OR Caregivers, Family OR Family Caregiver OR Informal Caregivers OR Caregiver, Informal OR Caregivers, Informal OR Informal Caregiver | 31210 |
|  | #6 | #4 OR #5 | 44195 |
|  | #7 | AB interview OR interviews OR experience OR qualitative | 545151 |
|  | #8 | #3 AND #6 AND 7 | 35 |
| **PsycInfo** | #1 | DE "Down's Syndrome" | 7131 |
|  | #2 | TI Down Syndrome OR Syndrome, Down OR mongolian OR 47,XY,+21 OR Trisomy G OR 47,XX,+21 OR Down's Syndrome OR Downs Syndrome OR Syndrome, Down's OR Trisomy 21 OR Trisomy 21, Mitotic nondisjunctive OR Down Syndrome, Partial Trisomy 21 OR Partial Trisomy 21 Down Syndrome OR Trisomy 21, Meiotic nondisjunctive | 7558 |
|  | #3 | #1 OR #2 | 9348 |
|  | #4 | DE "Caregivers" | 51205 |
|  | #5 | TI Caregiver OR Carers OR Carer OR Care Givers OR Care Giver OR Spouse Caregivers OR Caregiver, Spouse OR Caregivers, Spouse OR Spouse Caregiver OR Family Caregivers OR Caregiver, Family OR Caregivers, Family OR Family Caregiver OR Informal Caregivers OR Caregiver, Informal OR Caregivers, Informal OR Informal Caregiver | 27267 |
|  | #6 | #4 OR #5 | 46662 |
|  | #7 | AB interview OR interviews OR experience OR qualitative | 1141836 |
|  | #8 | #3 AND #6 AND #7 | 52 |
